# Intraduct papillary mucinous neoplasm of the pancreas: a tumour linked with IgG4-related disease?

**DOI:** 10.1136/jclinpath-2013-201516

**Published:** 2013-04-17

**Authors:** Adrian C Bateman, Emma L Culver, Matthew Sommerlad, Runjan Chetty

**Affiliations:** 1 Department of Cellular Pathology, University Hospital Southampton NHS Foundation Trust, Southampton, UK; 2 Translational Gastroenterology Unit and Nuffield Department of Medicine, University of Oxford, Oxford, Oxfordshire, UK; 3 Nuffield Department of Clinical Laboratory Sciences, University of Oxford, Oxford, Oxfordshire, UK

**Keywords:** PANCREAS, INFLAMMATION, PANCREATIC CANCER

## Abstract

**Objectives:**

Intraduct papillary mucinous neoplasm (IPMN) is a pancreatic tumour that is often associated with chronic pancreatitis (CP) in the surrounding pancreas. Type 1 autoimmune pancreatitis (AIP) is a fibro-inflammatory condition with characteristic histological features and prominent IgG4+ plasma cells and is part of the spectrum of IgG4-related disease (IgG4-RD). The aim of this study was to determine whether CP associated with pancreatic IPMN commonly represents AIP.

**Methods:**

We identified two consecutive ‘index’ cases of pancreatic IPMN during routine reporting in which the adjacent pancreas showed morphological features suggestive of AIP. These cases were investigated using the Boston criteria for the histopathological diagnosis of IgG4-RD and the HISORt criteria for the clinical and histopathological diagnosis of AIP. Using the same criteria, we proceeded to a clinical review of 12 extra cases of IPMN in which the tumour or the surrounding pancreas showed significant lymphoplasmacytic inflammation and/or fibrosis.

**Results:**

Both of the ‘index’ cases fulfilled the HISORt criteria for AIP and both had morphological features characteristic of IgG4-RD using the Boston criteria, although only one possessed features ‘highly suggestive of IgG4-RD’ after immunohistochemistry. Additionally, both ‘index’ cases had radiological features that could represent extrapancreatic manifestations. Review of the 12 additional cases of IPMN revealed no further examples showing co-existent AIP.

**Conclusion:**

While pancreatic IPMN and AIP may co-exist, most CP associated with IPMN does not represent AIP.

## Introduction

Intraduct papillary mucinous neoplasm (IPMN) of the pancreas is characterised by mucin production, cystic dilation of the pancreatic ducts and neoplastic intraductal papillary epithelial growth. Co-existent invasive carcinoma may be present. Chronic pancreatitis (CP) of the surrounding pancreas is a recognised feature. The CP associated with two consecutive cases of non-invasive IPMN identified during routine reporting was more florid than would usually be associated with a non-invasive pancreatic neoplasm and was suggestive of type 1 autoimmune pancreatitis (AIP). Type 1 AIP is the pancreatic manifestation of IgG4-related disease (IgG4-RD); a chronic and often multisystem fibroinflammatory condition associated with characteristic clinical, serological, radiological and histological features (the HISORt criteria for AIP, table 1), with the latter having been recently redefined (the Boston criteria).^1–3^ We reviewed the histological appearances, clinical and radiological data for these two index cases as well as for 12 further archival cases of non-invasive IPMN, in which the tumour or surrounding pancreas showed lymphoplasmacytic inflammation and/or fibrosis, to ensure that a diagnosis of AIP had not previously been overlooked.

**Table 1:** The HISORt criteria for the diagnosis of autoimmune pancreatitis^2^

1.	Histopathology—one or both criteria required	Characteristic appearances within biopsy or resection material*
		At least 10 IgG4-positive plasma cells per high power field within areas of lymphoplasmacytic infiltrate
2.	Imaging and serology—all three criteria required	Diffusely enlarged pancreas with delayed and ‘rim’ enhancement
		Irregular attenuated pancreatic duct
		Increased serum IgG4 concentration
		Unexplained pancreatic disease after a full clinical workup—including exclusion of cancer
		Raised serum IgG4 concentration and/or extrapancreatic organ involvement with increased numbers of tissue IgG4-positive plasma cells
3.	Response to steroid therapy—all three criteria required	Resolution or marked improvement in disease with steroid therapy

*This includes a lymphoplasmacytic infiltrate, ‘storiform’ fibrosis and obliterative phlebitis; the inflammatory cell infiltrate alone is not sufficient to meet this criterion.

## Methods

Two consecutive cases of non-invasive pancreatic IPMN associated with CP, with morphological features suggestive of AIP were identified at University Hospital Southampton NHS Foundation Trust (Southampton General Hospital). Both underwent appropriate work-up in order to confirm or exclude AIP/IgG4-RD. In association with collaborative research into IgG4-RD between Southampton and Oxford, 12 further cases (2008–11) of pancreatic IPMN from the Oxford Radcliffe Hospitals NHS Trust (John Radcliffe Hospital, Oxford, UK) in which the tumour or surrounding pancreas showed features raising the possibility of AIP/IgG4-RD, that is, lymphoplasmacytic inflammation and/or fibrosis, were reviewed with the specific aim of ensuring that a diagnosis of AIP/IgG4-RD had not been missed. These 12 cases were identified within the 23 consecutive IPMN resections performed in Oxford during this period.

All 14 cases underwent detailed histological examination and review of clinical records. Each case was assessed for the presence of morphological features that could indicate the presence of AIP/IgG4-RD, that is, lymphoplasmacytic inflammation, storiform fibrosis and obliterative phlebitis. An IgG4+ plasma cell count was performed within areas of lymphoplasmacytic inflammation, calculated as the mean IgG4+ plasma cell count per high power field (HPF; field diameter 0.6 mm) within the three HPF containing the greatest number of these cells. In cases in which the IgG4+ plasma cell count was 10 or more cells/HPF, IgG immunohistochemistry was performed in order to calculate an IgG4+/IgG+ ratio. The case records were examined for clinical, serological and/or radiological features that would support a diagnosis of AIP according to the HISORt criteria.

## Results

The histological and clinical features of each case are given in tables 2 and 3.

**Table 2:** Key histological features of the cases

Case	IPMN type	Inflammation in papillary stroma	Chronic pancreatitis surrounding tumour	Chronic pancreatitis distant to tumour	IgG4-RD associated morphology	IgG4+ plasma cells/HPF	Boston criteria for IgG4-RD met?
1	Intestinal	No	Yes	Yes	LPI/SF/OP	173 (65)*	Highly suggestive
2	Gastric	No	Yes	Yes	LPI/SF/OP	63 (26)*	Possible
3	Gastric	No	Yes	No	mLPI/F	3	No
4	Gastric	No	Yes	Yes	mLPI/F	0	No
5	Gastric	No	Yes	No	mLPI/F	1	No
6	Gastric	No	Yes	No	LPI/F	2	No
7	Gastric	No	Yes	Yes	LPI/F	4	No
8	Gastric	No	Yes	Yes	LPI/F	19 (14)*	No
9	Gastric	No	No	No	—	1	No
10	Intestinal	No	Yes	No	LPI/F	21 (12)*	No
11	Intestinal	No	Yes	Yes	LPI/F	9	No
12	Intestinal	Yes	Yes	No	LPI/F	4	No
13	Oncocytic	No	Yes	No	mLPI	11 (<1)*	No
14	Oncocytic	Yes	Yes	Yes	LPI/F	10 (<1)*	No

*The bracketed figures are the IgG4+/IgG+ ratio in cases in which the mean IgG4+ plasma cell count was greater than 10/HPF.

F, non-storiform fibrosis; IgG4-RD, IgG4-related disease; HPF, high power field; IPMN, intraduct papillary mucinous neoplasm; LPI, dense lymphoplasmacytic inflammation; mLPI, mild lymphoplasmacytic inflammation; OP,obliterative phlebitis; SF, storiform fibrosis.

**Table 3:** Key clinical features of the cases

Case	Demographics (sex, age (years))	Clinical presentation	Relevant history	Radiological characteristics	Serology	Possible other organ manifestations	Surgical treatment	Recurrence
1	Male, 75	AP, weight loss	DM	Focal mass HOP, PD dilated		Pulmonary nodules, retroperitoneal nodes, bulky ampulla	Whipple's	No
2	Male, 74	AP, weight loss	DM	Focal mass HOP, PD dilated		Solitary indeterminate lung nodule, peripancreatic nodes	Total pancreatectomy	No
3	Male, 79	AP		Focal mass HOP and neck, PD dilated, stricture PD			Total pancreatectomy	No
4	Male, 75	AP	DM	Focal mass HOP, PD dilated			Whipple's and postoperative chemotherapy	Yes
5	Male, 66	AP	DM, hypothyroid	Focal mass HOP, pancreatitis, PV thrombus			Whipple's	Yes
6	Male, 67	AP		Focal mass HOP, PD dilated, pancreatitis			Whipple's	No
7	Female, 63	AP, weight loss		Focal mass HOP and uncinate, PD dilated, atrophy			Whipple's	No
8	Female, 57	AP		Focal mass BOP, PD dilated			Whipple's and postoperative chemotherapy	No
9	Male, 72	None		Focal mass HOP, PD dilated			Total pancreatectomy and splenectomy	No
10	Male, 77	Jaundice	Asthma, atopy	Focal mass uncinate, PD dilated, PV thrombosis	sIgG8.87 d/l, sIgG4 0.27 g/l, ANA negative		Whipple's and postoperative chemotherapy	Yes
11	Male, 59	Steatorrhoea		Focal mass HOP, PD dilated			Whipple's	No
12	Male 58	None	Opthalmoplegia (no cause)	Focal mass HOP, PD dilated, atrophy			Total pancreatectomy	No
13	Female, 59	AP		Focal mass TOP, PD dilated			Distal pancreatectomy	Yes
14	Male, 76	None		Focal mass HOP			Whipple's	No

ANA, antinuclear antibody; AP, abdominal pain; BOP, body of pancreas; DM, type 2 diabetes mellitus; HOP, head of pancreas; PD, pancreatic duct; PV  portal vein; TOP, tail of pancreas.

### Histological review

**Figure 1:**
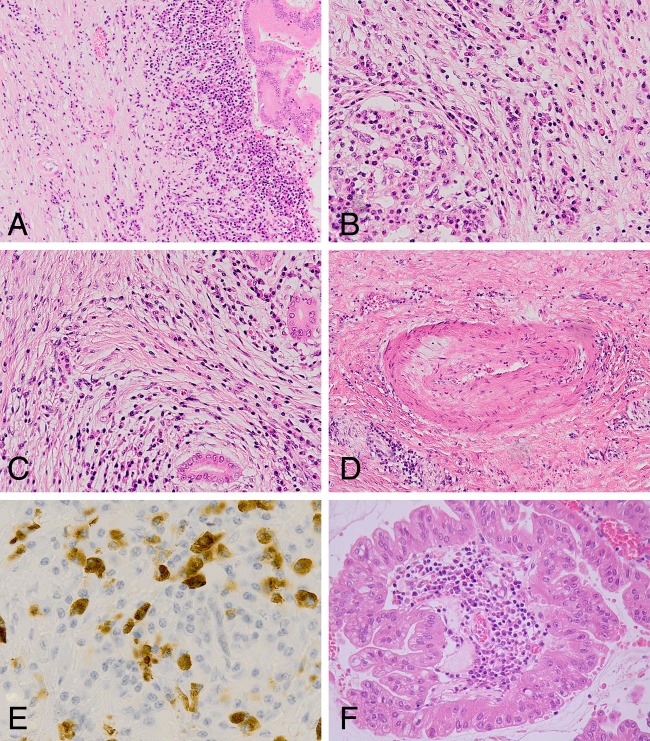
Histological appearances of case 1 (A–E) and case 14 (F). (A) Medium power view showing edge of intraduct papillary mucinous neoplasm (IPMN) with chronic inflammation and fibrosis extending into the adjacent pancreas. (B) Lymphoplasmacytic inflammation adjacent to a surviving islet of Langerhans within the distant pancreas. (C) Storiform fibrosis. (D) Obliterative venulitis. (E) IgG4 immunohistochemistry revealing numerous IgG4+ plasma cells within the inflammatory infiltrate. (F) Oncocytic variant of IPMN showing prominent lymphoplasmacytic inflammation within a papillary core.

The two ‘index’ cases (cases 1 and 2) showed CP immediately surrounding the IPMN as well as within the distant pancreas. The characteristic morphological features of lymphoplasmacytic inflammation, storiform fibrosis and obliterative phlebitis were present in both cases. Both cases also showed very prominent IgG4+ plasma cell numbers within the infiltrate (mean of 173 and 63 IgG4+ plasma cells/HPF, respectively) while the IgG4+/IgG ratio was significantly elevated in only case 1 (figure 1). Eleven of the remaining 12 cases showed CP surrounding the tumour but only five showed CP within the distant pancreas. In the cases showing CP, the degree of lymphoplasmacytic inflammation was mild in four cases, and while fibrosis was present this did not show a storiform pattern. Obliterative phlebitis was not identified in any of these 12 cases. Four of the 12 cases showed an IgG4+ plasma cell count of 10 or more cells/HPF but the IgG4+/IgG+ ratio was less than 40% (cut-off defined in the Boston criteria). Two cases (cases 12 and 14) contained chronic inflammation within the papillary stroma, and this was particularly prominent within one of the two oncocytic IPMN (figure 1). None of the cases showed features suggestive of type 2 AIP, for example, granulocytic epithelial lesions.

### Clinical review

The 14 patients with IPMN were predominantly man in the sixth to seventh decades of life and presented with abdominal pain and weight loss rather than jaundice. All had a focal mass on imaging, most involved the head of the pancreas and were associated with a dilated pancreatic duct. Only one patient had undergone serum IgG4 measurement as AIP was in the differential diagnosis. No patient received a trial of corticosteroids. Case 1 had indeterminate pulmonary nodules and prominent retroperitoneal lymph nodes and case 2 had a solitary indeterminate pulmonary nodule and peri-pancreatic nodes (table 3). These may be suggestive of extra-pancreatic organ involvement. None of the 14 patients had a history of chronic excess alcohol consumption.

### Criteria for AIP

On the basis of histological and clinical review, case 1 had features ‘histologically highly suggestive of IgG4-RD’ according to the Boston criteria and fulfilled the HISORt criteria for AIP. Using the same guidelines, case 2 possessed histological features suggestive of IgG4-RD but without an IgG4+/IgG+ ratio over 40% that is ‘mandatory’ for a diagnosis of IgG4-RD according to the Boston criteria, although the morphological characteristics and IgG4+ plasma cells met the HISORt criteria for AIP. None of the remaining cases possessed histological or clinical features that fulfilled either set of diagnostic criteria.

## Discussion

Pancreatic neoplasms are commonly associated with CP in the immediately surrounding parenchyma. Distant pancreatic tissue may also show CP if there has been obstruction of the main pancreatic duct, and this feature can be particularly marked in invasive tumours such as ductal adenocarcinoma. The severity and extent of CP associated with non-invasive IPMN is usually less than that seen classically with ductal adenocarcinoma. Therefore, the presence of widespread severe CP within the two ‘index’ cases in this study was striking and unexpected.

Histological examination of the two ‘index’ cases revealed morphological features (lymphoplasmacytic inflammation, storiform fibrosis and obliterative phlebitis) characteristic of IgG4-RD according to the Boston criteria.^3^ While none of these features are specific for IgG4-RD, if all are present they are highly suggestive of the condition when taken in an appropriate clinical context and in association with a raised tissue IgG4+ plasma cell count. A raised tissue IgG4+ plasma cell count is also not specific for IgG4-RD (table 4) but is required in addition to the morphological features in order to fulfil the Boston criteria. Both of the index cases possessed tissue IgG4+ plasma cell counts of more than 50 cells/HPF—a threshold for diagnosis set for surgical specimens of the pancreas by the Boston criteria. However, the additional mandatory requirement of an IgG4+/IgG+ ratio of over 40% was met by only case 1. Interestingly, both case 1 and 2 had radiological features that may suggest extra-pancreatic involvement—namely pulmonary nodules of indeterminate nature and enlarged lymph nodes—although there were no histological specimens from these sites to support this. While all but one of the remaining 12 cases showed CP, the morphological features were not typical of AIP. In particular, storiform fibrosis and obliterative venulitis were not seen. Furthermore, none of these cases were associated with a tissue IgG4+ plasma cell count of more than 50 such cells/HPF or an IgG4+/IgG ratio of over 40%. None of these cases had radiological features suggestive of IgG4-RD and we believe that the CP in these cases was obstructive in nature, that is, secondary to the IPMN. These findings support the view that the minimum cut-off of more than 10 IgG4+ plasma cells/HPF, as used in the HISORt criteria, is not sufficiently high to be specific for a diagnosis of IgG4-RD. The minimum cut-off for IgG4+ plasma cells suggested by the Boston criteria when considering a diagnosis of IgG4-RD is over 50 such cells/HPF—a criterion met by only our first two cases.^3^ Furthermore, none of cases 3–14 possessed more than one of the three characteristic morphological criteria for IgG4-RD. In particular, all but one of these 12 cases showed lymphoplasmacytic inflammation, but this was mild in four cases. Ten of the same 12 cases showed fibrosis but this was not storiform in appearance.

**Table 4:** Conditions that may show prominent tissue IgG4+ plasma cells in the absence of IgG4-RD

Site	Condition
Oral cavity^4^	Epulis plasmacellularis
	Radicular cyst
	Carcinoma
Colon^4^	Diverticulitis
Synovium^4^	Rheumatoid arthritis
Skin^4^	Inflammatory skin conditions
Pancreas^5^	Carcinoma
Lymph nodes	Castleman's disease^6^
	Follicular hyperplasia^6^
	Interfollicular plasmacytosis^6^
	Lymphoma^7^
Colon^8^	Inflammatory bowel disease
Lung^9^	Rosai–Dorfman disease
Aorta^10^	Peri-aortitis

IgG4-RD, IgG4-related disease.

Our review has confirmed one case ‘histologically highly suggestive of AIP/IgG4-RD’ and one further case of possible AIP/IgG4-RD, associated with pancreatic IPMN. These were identified during routine histopathology reporting due to morphological features within the adjacent and distant pancreas. However, our clinical and histological review of 12 additional cases has suggested that an association between pancreatic IPMN and the presence of AIP/IgG4-RD is not common. It does, however, highlight the importance of careful study of the ‘background’ pancreas within surgical specimens removed for pancreatic neoplasia as well as purely for CP. IgG4 immunohistochemistry is an important component of this assessment, but the tissue IgG4+ count (and IgG4+/IgG+ ratio) must always be interpreted in the context of the morphological features of a case and the clinical scenario. Recognition of IgG4-RD is essential as the condition is potentially multisystem in nature and may be underdiagnosed, especially if the clinical presentation is atypical. Furthermore, the disease usually responds well to corticosteroid therapy, but relapse is common and therefore follow-up in a specialist centre is recommended.^1^

**Take home messages**

- IPMN of the pancreas may be associated with morphological features in the surrounding pancreas that raise the possibility of type 1 AIP/IgG4-RD.
- IPMN of the pancreas may be associated with AIP/IgG4-RD but the majority of cases showing histological evidence of CP in the adjacent tissue do not represent examples of AIP/IgG4-RD.
- A histological diagnosis of IgG4-RD requires the presence of key morphological characteristics—lymphoplasmacytic inflammation, storiform fibrosis and obliterative phlebitis—together with prominent IgG4+ plasma cell numbers and an IgG4+/IgG+ plasma cell ratio of over 40%.
- Despite the sometimes very characteristic histological features of IgG4-RD, it is always essential to interpret histological findings in the overall clinical context of the case, for example, through the use of guidelines such as the HISORt criteria.
